# Superresolution Microscopy Reveals Distinct Phosphoinositide Subdomains Within the Cilia Transition Zone

**DOI:** 10.3389/fcell.2021.634649

**Published:** 2021-04-30

**Authors:** Sarah E. Conduit, Elizabeth M. Davies, Alex J. Fulcher, Viola Oorschot, Christina A. Mitchell

**Affiliations:** ^1^Cancer Program, Monash Biomedicine Discovery Institute, Department of Biochemistry and Molecular Biology, Monash University, Clayton, VIC, Australia; ^2^Monash Micro Imaging, Monash University, Clayton, VIC, Australia; ^3^Monash Ramaciotti Centre for Structural Cryo-Electron Microscopy, Monash University, Clayton, VIC, Australia

**Keywords:** primary cilia, transition zone, INPP5E, phosphoinositides, superresolution microscopy

## Abstract

Primary cilia are evolutionary conserved microtubule-based organelles that protrude from the surface of most mammalian cells. Phosphoinositides (PI) are membrane-associated signaling lipids that regulate numerous cellular events via the recruitment of lipid-binding effectors. The temporal and spatial membrane distribution of phosphoinositides is regulated by phosphoinositide kinases and phosphatases. Recently phosphoinositide signaling and turnover has been observed at primary cilia. However, the precise localization of the phosphoinositides to specific ciliary subdomains remains undefined. Here we use superresolution microscopy (2D stimulated emission depletion microscopy) to map phosphoinositide distribution at the cilia transition zone. PI(3,4,5)P_3_ and PI(4,5)P_2_ localized to distinct subregions of the transition zone in a ring-shape at the inner transition zone membrane. Interestingly, the PI(3,4,5)P_3_ subdomain was more distal within the transition zone relative to PtdIns(4,5)P_2_. The phosphoinositide effector kinase pAKT(S473) localized in close proximity to these phosphoinositides. The inositol polyphosphate 5-phosphatase, INPP5E, degrades transition zone phosphoinositides, however, studies of fixed cells have reported recombinant INPP5E localizes to the ciliary axoneme, distant from its substrates. Notably, here using live cell imaging and optimized fixation/permeabilization protocols INPP5E was found concentrated at the cilia base, in a distribution characteristic of the transition zone in a ring-shaped domain of similar dimensions to the phosphoinositides. Collectively, this superresolution map places the phosphoinositides in situ with the transition zone proteins and reveals that INPP5E also likely localizes to a subdomain of the transition zone membrane, where it is optimally situated to control local phosphoinositide metabolism.

## Introduction

The small hair-like sensory organelle the primary cilium is a critical regulator of cell biology (Bangs and Anderson, 2017). *In vivo* most cell types exhibit a single primary cilium that projects from the surface and detects external cues. The essential role these organelles play in development and homeostasis is highlighted by the ciliopathy syndromes, caused by mutations in key ciliary genes, which result in severe phenotypes including embryonic lethality, exencephaly, blindness, polycystic kidneys and mental retardation, among others (Wheway and Mitchison, 2019). The ability of the primary cilium to concentrate signaling molecules and sample the environment makes it an ideal hub for signal transduction. Indeed Hedgehog, Wnt, planar cell polarity, receptor tyrosine kinases and G protein-coupled receptors transduce signals via the cilium (Huangfu et al., 2003; Schneider et al., 2005; Simons et al., 2005; Corbit et al., 2008).

Primary cilia are microtubule-based structures anchored by a modified mother centriole, known as the basal body. The axoneme is made up of 9 microtubule doublets (in a 9 + 0 arrangement) which extend from the basal body. The ciliary membrane covers the axoneme and is continuous with the plasma membrane but enriched with a distinctive protein and lipid complement. The transition zone is the region at the base of the axoneme distal to the basal body which acts a gate governing the entry and exit of molecules to the cilium (Gonçalves and Pelletier, 2017). This zone consists of three multi-protein complexes MKS, NPHP and CEP290 that form part of the ciliary diffusion barrier (Chih et al., 2011; Garcia-Gonzalo et al., 2011; Sang et al., 2011), however, the molecular mechanisms of barrier function remain incompletely understood. These complexes consist of transmembrane, membrane-associated and cytosolic proteins which are dependent upon each other for localization to the transition zone.

Phosphoinositides (PIs) are low abundance membrane lipids that play diverse signaling roles and have been recently identified in the ciliary membrane and transition zone (Chavez et al., 2015; Garcia-Gonzalo et al., 2015; Park et al., 2015; Conduit et al., 2017; Dyson et al., 2017) (reviewed in Conduit and Vanhaesebroeck (2020)). PIs consist of a fatty acid backbone anchored in the membrane with a cytosol facing inositol head group that can be decorated by phosphorylation of the 3-, 4- and 5-positions, producing six phosphorylated species with distinct signaling functions. PI(4)P, PI(4,5)P_2_, PI(3,4,5)P_3_ and PI(3,4)P_2_ localize to the primary cilium with PI(4,5)P_2_, PI(3,4,5)P_3_ and PI(3,4)P_2_ identified at the transition zone (Chavez et al., 2015; Garcia-Gonzalo et al., 2015; Park et al., 2015; Conduit et al., 2017; Dyson et al., 2017). Notably many transition zone proteins contain putative PI binding C2 and B9 domains but whether these proteins actually bind PIs *in vivo* at cilia has not been confirmed (Dowdle et al., 2011; Garcia-Gonzalo and Reiter, 2012; Remans et al., 2014). Established PI-binding effectors, such as the pleckstrin homology (PH) domain-containing activated form of the serine threonine kinase AKT also localize between the basal body and the axoneme, possibly at the transition zone (Zhu et al., 2009; Schneider et al., 2010; Higginbotham et al., 2013; Suizu et al., 2016; Conduit et al., 2017). Accumulation of PI(4,5)P_2_ and PI(3,4,5)P_3_ at the transition zone is associated with disruption of the transition zone protein complexes and compromised diffusion barrier function (Dyson et al., 2017). This suggests the PIs are critical organizers of transition zone architecture, however, the precise interactions and structural arrangement of the PIs within the transition zone protein network have not been reported to date.

The transition zone PIs are regulated by the inositol polyphosphate 5-phosphatase INPP5E (Conduit et al., 2017; Dyson et al., 2017) which dephosphorylates PI(4,5)P_2_ and PI(3,4,5)P_3_ to produce PI(4)P and PI(3,4)P_2_ respectively (Kisseleva et al., 2000; Kong et al., 2000). INPP5E mutations cause two ciliopathy syndromes, Joubert syndrome and MORM (Bielas et al., 2009; Jacoby et al., 2009). In mice, *Inpp5e* deletion is embryonic lethal with classical cilia dysfunction phenotypes and repression of cilia-dependent Hedgehog signaling (Jacoby et al., 2009; Chavez et al., 2015; Garcia-Gonzalo et al., 2015; Dyson et al., 2017). *Inpp5e*-null cells show elevated transition zone PI(4,5)P_2_ and PI(3,4,5)P_3_ signals and defective transition zone function (Dyson et al., 2017). Multiple studies have reported INPP5E localizes to the ciliary axoneme, a distribution that is disrupted by the MORM syndrome mutation (Jacoby et al., 2009; Garcia-Gonzalo et al., 2011; Humbert et al., 2012; Roberson et al., 2015; Slaats et al., 2016; Conduit et al., 2017; Dyson et al., 2017; Goetz et al., 2017; Qiu et al., 2021). INPP5E localization to the axoneme is dependent on a functional transition zone and a network of the cilia associated proteins ARL13B, PDE6D and CEP164 (Garcia-Gonzalo et al., 2011; Humbert et al., 2012; Roberson et al., 2015; Slaats et al., 2016; Goetz et al., 2017; Qiu et al., 2021). However, this raises the question of how a 5-phosphatase in the cilia axoneme can access and tightly control its substrates at the transition zone. Perhaps INPP5E transiently passes through the transition zone *en route* to the axoneme and in the process hydrolyses the PIs, however, such transient localization is not easily detected in fixed cells. Consistent with this contention, in a small subset of fixed cells, low level transition zone INPP5E has been observed (Dyson et al., 2017), and in photoreceptors that have a highly modified cilium, INPP5E localizes to the connecting cilium which corresponds to the transition zone (Sharif et al., 2021). Alternatively, it is possible that standard immunofluorescence (IF) techniques used to date do not accurately preserve and represent the correct INPP5E localization.

The transition zone is ∼250–300 nm in diameter, which is similar to the resolution limit of confocal microscopy, meaning all components appear to co-localize as a single punctum at this site using confocal microscopy (Yang et al., 2015). Therefore, higher resolution imaging techniques are required to elucidate the molecular organization of protein and PI components within the transition zone, a critical step to understand how the transition zone diffusion barrier operates. Immunoelectron microscopy has provided some insights into cilia ultra-structure (Deane et al., 2001; Craige et al., 2010), but cannot be used to directly visualize PIs. Expression of PI binding biosensors enables detection via electron microscopy but is also limited by potential lipids sequestration artifacts, interferes with function and does not always accurately reflect PI localization (Hammond and Balla, 2015). Superresolution 2D stimulated emission depletion (STED) microscopy provides 50 nm resolution and has been used to begin to map the transition zone protein architecture (Yang et al., 2015). Superresolution imaging of the transition zone has revealed that many of the protein components exhibit a hollow ring-shaped domain, surrounding other components occupying a solid domain in the center with a similar width to the microtubule axoneme. These proteins also localize to distinct domains in the proximal-distal axis of the transition zone (Yang et al., 2015).

Superresolution STED microscopy provides the ideal methodology to map the localization of PIs, their regulatory enzymes and effectors in the context of the transition zone protein network, overcoming the resolution limit of light microscopy and technical limitations of electron microscopy. Here, we mapped the transition zone localization of the PIs PI(4,5)P_2_ and PI(3,4,5)P_3_, the PI effector pAKT(S473), and the PI regulator INPP5E using STED microscopy and placed these transition zone components within the context of the recently described transition zone protein network.

## Results

### Phosphoinositides Localize to the Cilia Transition Zone

We have shown PI(4,5)P_2_ and PI(3,4,5)P_3_ localize to the cilia transition zone (Conduit et al., 2017; Dyson et al., 2017), however, their precise localization within the complex architecture created by the transition zone protein components is unknown. This is in part due to the resolution of confocal microscopy, which has been used for all studies of ciliary PIs to date (Chavez et al., 2015; Garcia-Gonzalo et al., 2015; Park et al., 2015; Conduit et al., 2017; Dyson et al., 2017) (reviewed in Conduit and Vanhaesebroeck (2020)) and is limited to ∼250 nm, approximately the same size as the diameter of the transition zone (Yang et al., 2015). 2D STED microscopy is a superresolution imaging technique that reaches 50 nm resolution for cells (Hell and Wichmann, 1994; Klar et al., 2000; Donnert et al., 2007), making it ideal to resolve the transition zone architecture. Indeed, STED imaging was recently used to map the localization of several transition zone proteins (Yang et al., 2015). To obtain the highest resolution in the plane parallel to the axoneme and maintain resolution in the perpendicular plane, imaging was undertaken using a light path perpendicular to the axoneme using the well characterized ciliated human retinal pigment epithelial cell line, hTERT-retinal pigment epithelial 1 (RPE1), as reported previously (Yang et al., 2015). The majority of hTERT-RPE1 cilia form parallel to the imaging plane making this approach possible.

**FIGURE 1:**
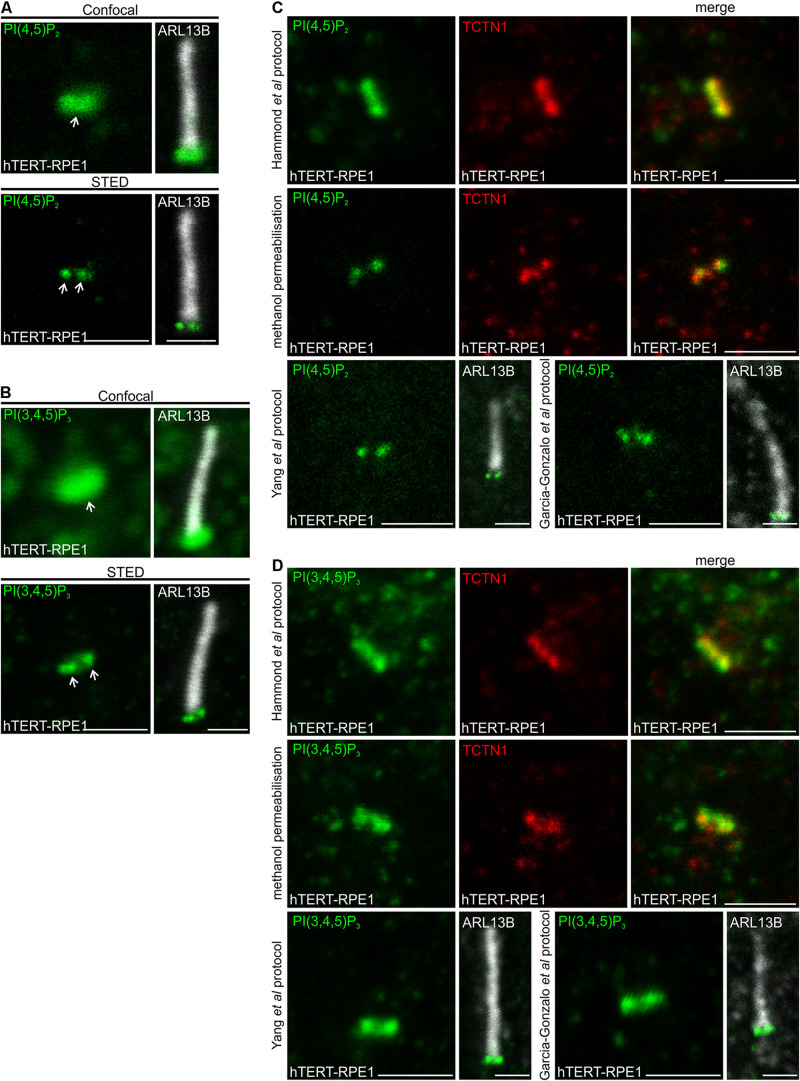
PI(4,5)P_2_ and PI(3,4,5)P_3_ localize to the transition zone in ciliated hTERT-RPE1 cells. **(A,B)** Ciliated hTERT-RPE1 cells were immunostained with **(A)** PI(4,5)P_2_ or **(B)** PI(3,4,5)P_3_ (green) and ARL13B (grayscale) antibodies and imaged by confocal and STED microscopy. Right panels show merged image at lower magnification. Arrows indicate transition zone PI(4,5)P_2_ signals, bar indicates 1 μm. **(C,D)** Ciliated hTERT-RPE1 cells were immunostained with **(C)** PI(4,5)P_2_ (green) or **(D)** PI(3,4,5)P_3_ (green) and TCTN1 (red) or ARL13B (grayscale) antibodies using the Hammond et al. (2009) Golgi protocol, the methanol permeabilization protocol, the Yang et al. (2015) or Garcia-Gonzalo et al. (2011) protocol and imaged by confocal microscopy, bar indicates 1 μm.

The PI distribution observed via STED microscopy was first compared to confocal resolution in the same cell. A single PI(4,5)P_2_ punctum was observed at the transition zone by confocal microscopy. In contrast, STED imaging enabled the detection of two distinct intensity peaks, characteristic of a cross section through a ring-shape structure (Figure 1A). The distribution of PI(3,4,5)P_3_, which is generated from PI(4,5)P_2_ by phosphoinositide 3-kinases (PI3Ks), was evaluated using STED imaging and also exhibited two distinct signals at the transition zone (Figure 1B), suggesting these related PIs may co-localize to the same transition zone subdomain.

Several studies have reported PI(4,5)P_2_, PI(4)P and PI(3,4,5)P_3_ localization to cilia but with different distributions, possibly due to differences in detection, fixation and/or staining techniques (Chavez et al., 2015; Garcia-Gonzalo et al., 2015; Conduit et al., 2017; Dyson et al., 2017; Phua et al., 2017; Conduit and Vanhaesebroeck, 2020). For example, Chavez et al. (2015) reported that PI(4,5)P_2_ localizes to the ciliary axoneme, concentrated at the ciliary tip. PI(4,5)P_2_ was also observed at the proximal end of the axoneme by Garcia-Gonzalo et al. (2015) and Phua et al. (2017). In contrast, we have consistently observed PI(4,5)P_2_ concentrated at the transition zone at the base of the cilium in multiple cell types (Conduit et al., 2017; Dyson et al., 2017). The PI fatty acid back bone localizes to the membrane whilst the inositol ring, which is reversibly phosphorylated, faces the cytosol and as such these lipids can be extracted from the membrane by detergent or during fixation. To optimize superresolution imaging, we performed a comprehensive comparison of the PI distribution obtained using multiple different staining techniques based on previous reports of ciliary PI and transition zone component detection, using validated PI antibodies (Hammond et al., 2006, 2009; Yip et al., 2008). We have reported that the transition zone PI(4,5)P_2_ antibody signal is ablated by Neomycin which blocks the lipid and the transition zone PI(3,4,5)P_3_ antibody signal is reduced by pan-PI3K inhibition (Dyson et al., 2017). PI(4,5)P_2_ was examined using the validated “Golgi” staining protocol developed by Hammond et al. (2009), and revealed PI(4,5)P_2_ localized to the ciliary transition zone, co-localizing with the transition zone marker TCTN1 (Figure 1C). A consistent transition zone PI(4,5)P_2_ distribution at the cilia base was also observed using a standard IF protocol with methanol permeabilization and protocols reported by Yang et al. (2015) and Garcia-Gonzalo et al. (2011) for detection of transition zone components (Figure 1C). We then assessed the ciliary localization of PI(3,4,5)P_3_ using these staining techniques. PI(3,4,5)P_3_ exhibited a similar distribution at the transition zone (co-localizing with TCTN1) via all staining protocols utilized (Figure 1D). Finally, PI(4)P has been reported to localize to the axoneme (Chavez et al., 2015; Garcia-Gonzalo et al., 2015). We tested the Hammond et al. (2009) “Golgi” staining protocol as it is the most well-established method for intracellular PI detection by indirect IF. PI(4)P signals were detected in a distribution resembling the Golgi (Supplementary Figure 1A) as expected (Hammond et al., 2009), however, we failed to detect any cilia associated PI(4)P signals using this method for unknown reasons.

Chavez et al. (2015) observed both PI(4,5)P_2_ and PI(4)P in the cilia axoneme in neural stem cells, contrasting with our findings here and as we reported previously (Conduit et al., 2017; Dyson et al., 2017). Therefore, we used the protocol reported by Chavez et al. (2015), which includes paraformaldehyde (PFA) fixation and 0.3% Triton X-100 permeabilization, to assess cilia PIs in MEFs. In contrast to the Chavez et al. (2015) report, here under these conditions PI(4,5)P_2_ was detected at the transition zone, whereas PI(4)P and PI(3,4,5)P_3_ were not detected using this method (Supplementary Figure 1B). The transition zone PI(4,5)P_2_ signal was also observed using these staining conditions in hTERT-RPE1 cells (Supplementary Figure 1C). The reasons for the distinct PI(4,5)P_2_ localization and inability to detect ciliary PI(4)P under the same staining conditions as reported previously are unclear, but may relate to the use of different antibodies or subtle changes in the duration or conditions of fixation and/or permeabilization.

### PI(4,5)P_2_ and PI(3,4,5)P_3_ Localize to Distinct Subdomains of the Transition Zone

**FIGURE 2:**
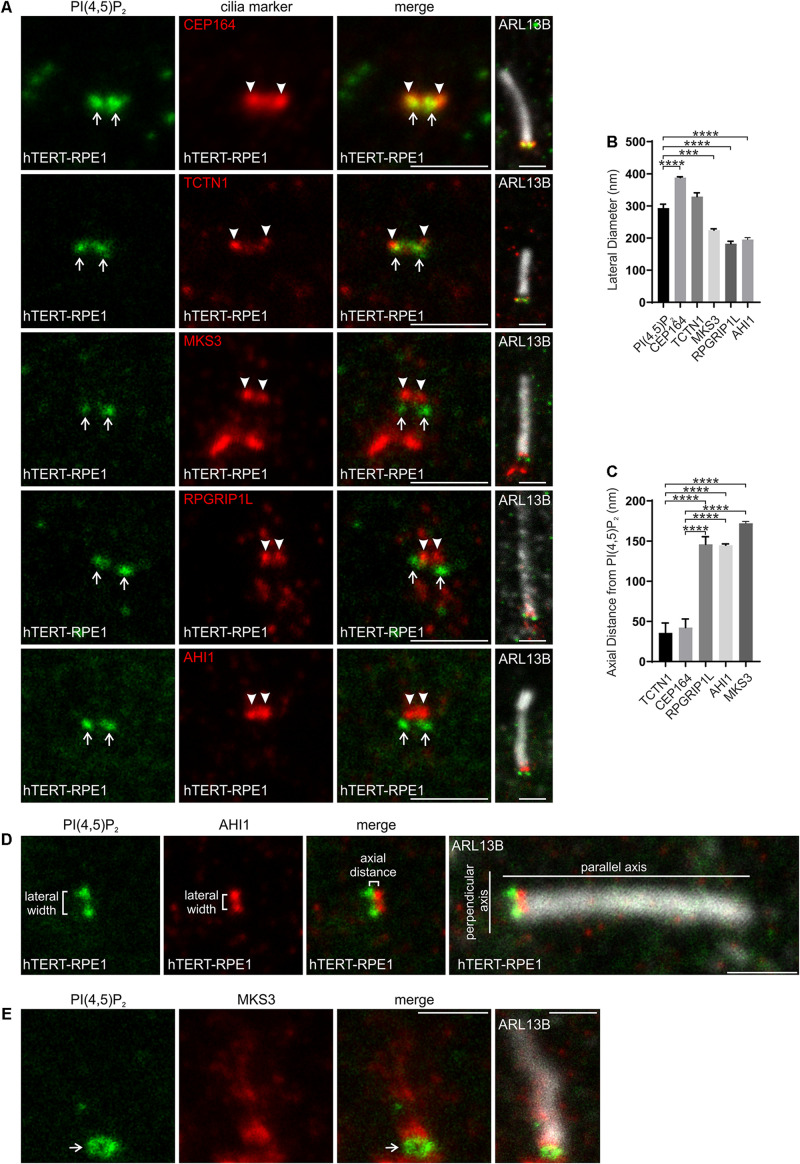
PI(4,5)P_2_ localizes to a specific subdomain of the transition zone. **(A)** Ciliated hTERT-RPE1 cells were immunostained with PI(4,5)P_2_ (green), ARL13B (grayscale) and CEP164, TCTN1, MKS3, RPGRIP1L or AHI1 (red) antibodies and imaged by STED microscopy (confocal resolution image of the ARL13B stained axoneme is shown). Right panels show merged image at lower magnification. Arrows indicate transition zone PI(4,5)P_2_ signals, arrow heads indicate transition zone protein localization, bar indicates 1 μm. **(B)** Graph shows the lateral diameter between the highest intensity points of the PI(4,5)P_2_ or cilia protein marker puncta perpendicular to the plane of the axoneme. Bars represent mean ± SEM, *n* = 3 independent experiments, ≥30 cilia imaged per experiment and all cilia with two distinct PI(4,5)P_2_ or cilia marker protein puncta measured, statistical significance was determined using one-way ANOVA (*p* < 0.0001) followed by Tukey’s post hoc test ***p < 0.001, ****p < 0.0001. **(C)** Graph shows the axial distance between the highest intensity point of the PI(4,5)P_2_ signal and each cilia marker protein signal parallel to the plane of the axoneme. Bars represent mean ± SEM, *n* = 3 independent experiments, ≥ 30 cilia imaged per experiment and all cilia with distinct PI(4,5)P_2_ or cilia marker protein puncta measured, statistical significance was determined using one-way ANOVA (*p* < 0.0001) followed by Tukey’s post hoc test, ****p < 0.0001. **(D)** Representative image showing the method used for lateral diameter and axial distance measurements, bar indicates 1 μm. **(E)** Ciliated hTERT-RPE1 cells were immunostained with PI(4,5)P_2_ (green), MKS3 (red) and ARL13B (grayscale) antibodies and imaged by STED microscopy (confocal resolution image of the ARL13B stained axoneme is shown). Right panel shows merged image at lower magnification. Arrow indicates ring shaped transition zone PI(4,5)P_2_ morphology, bar indicates 1 μm.

We next mapped the localization of PI(4,5)P_2_ at the transition zone in hTERT-RPE1 cells relative to transition zone proteins TCTN1, MKS3, RPGRIP1L and AHI1, components of the MKS and NPHP complexes (Gong et al., 2018), and distal appendage protein CEP164 to mark the proximal boundary of the transition zone (Yang et al., 2015). STED resolution imaging was performed for the PI and transition zone protein components and overlayed on a confocal resolution image of the ARL13B stained axoneme (Figure 2A). PI(4,5)P_2_ exhibited a wider lateral distribution than MKS3, RPGRIP1L or AHI1, a similar width compared to TCTN1 and a significantly narrower domain than CEP164 (Figures 2A,B,D). RPGRIP1L localized toward the center of the transition zone (Yang et al., 2015). Note, we also observed MKS3 staining proximal to the transition zone which may correspond to the basal body given MKS3 was previously shown to localize to olfactory sensory neuron dendritic knobs, which contain the olfactory cilia basal bodies (Pluznick et al., 2011). TCTN1 contains a signal peptide but no extracellular domains and functions cell autonomously suggesting it is closely associated with the transition zone membrane (Garcia-Gonzalo et al., 2011). PI(4,5)P_2_ localization relative to the transition zone proteins (Figures 2A,B) is consistent with a localization within the inner leaflet of the transition zone membrane. In the axial plane, parallel to the axis of the axoneme, PI(4,5)P_2_ was more proximal to the cilia base than MKS3, RPGRIP1L and AHI1 and closest (less than 50 nm, the limit of STED resolution) to TCTN1 (Figures 2A,C,D). Interestingly, both PI(4,5)P_2_ and TCTN1 were < 50 nm from CEP164 in the axial plane, which represents the most proximal level of the transition zone (Figures 2A,C,D). In some cells where the cilium was located at an angle to the light path, PI(4,5)P_2_ exhibited a hollow ring-shape at the transition zone (Figure 2E).

**FIGURE 3:**
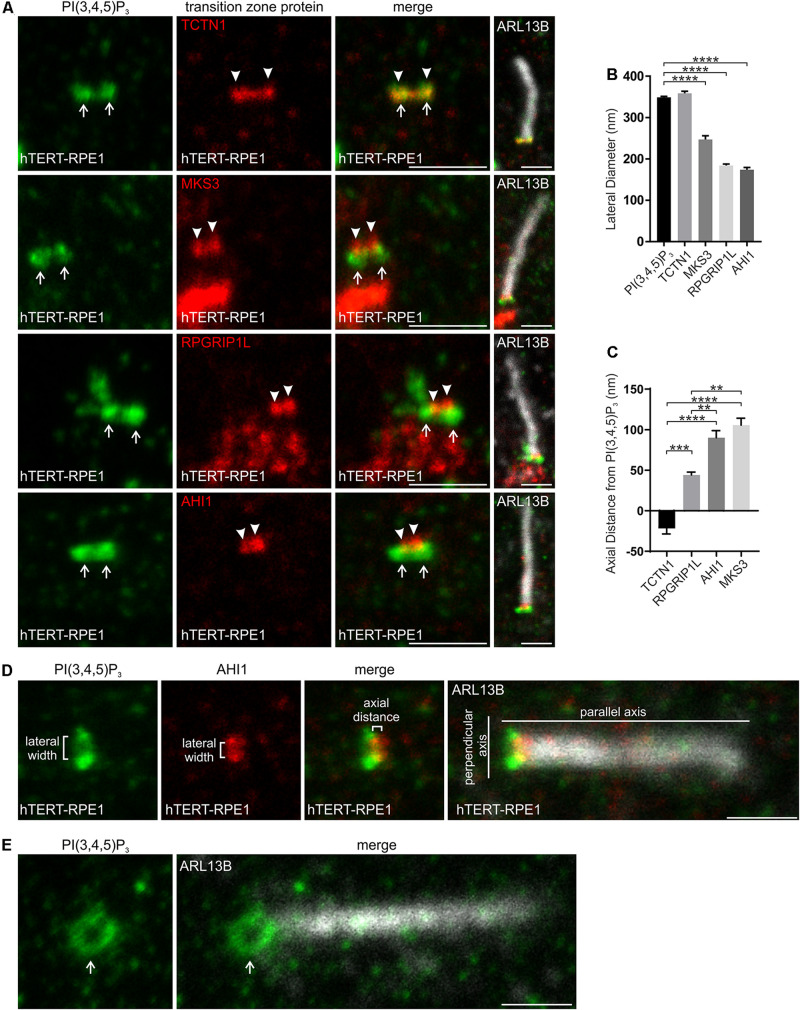
PI(3,4,5)P_3_ localizes to a specific subdomain of the transition zone. **(A)** Ciliated hTERT-RPE1 cells were immunostained with PI(3,4,5)P_3_ (green), ARL13B (grayscale) and TCTN1, MKS3, RPGRIP1L or AHI1 (red) antibodies and imaged by STED microscopy (confocal resolution image of the ARL13B stained axoneme is shown). Right panels show merged image at lower magnification. Arrows indicate transition zone PI(3,4,5)P_3_ signal, arrow heads indicate transition zone protein localization, bar indicates 1 μm. **(B)** Graph shows the lateral diameter between the highest intensity points of the PI(3,4,5)P_3_ or transition zone protein puncta perpendicular to the plane of the axoneme. Bars represent mean ± SEM, *n* = 3 independent experiments, 30 cilia imaged per experiment and all cilia with two distinct PI(3,4,5)P_3_ or transition zone protein puncta measured, statistical significance was determined using one-way ANOVA (*p* < 0.0001) followed by Tukey’s post hoc test, ****p < 0.0001. **(C)** Graph shows the axial distance between the highest intensity point of the PI(3,4,5)P_3_ signal and each transition zone protein signal parallel to the plane of the axoneme. Bars represent mean ± SEM, *n* = 3 independent experiments, 30 cilia imaged per experiment and all cilia with distinct PI(3,4,5)P_3_ or transition zone protein puncta measured, statistical significance was determined using one-way ANOVA (*p* < 0.0001) followed by Tukey’s post hoc test, **p < 0.01, ***p < 0.001, ****p < 0.0001. **(D)** Representative image showing the method used for the lateral diameter and axial distance measurements, bar indicates 1 μm. **(E)** Ciliated hTERT-RPE1 cells were immunostained with PI(3,4,5)P_3_ (green) and ARL13B (grayscale) antibodies and imaged by STED microscopy (confocal resolution image of the ARL13B stained axoneme is shown). Arrow indicates ring shaped transition zone PI(3,4,5)P_2_ morphology, bar indicates 1 μm.

The lateral width of the PI(3,4,5)P_3_ signal was similar to TCTN1, but significantly greater than MKS3, RPGRIP1L and AHI1 (Figures 3A,B,D), suggesting PI(3,4,5)P_3_ also localizes to the inner leaflet of the transition zone membrane. In the axial plane PI(3,4,5)P_3_ was more proximal than RPGRIP1L, MKS3 or AHI1 and less than 50 nm distal to TCTN1 (Figures 3A,C,D). Ring-shaped PI(3,4,5)P_3_ was also observed in cells with cilia at an angle to the plane of imaging (Figure 3E). Therefore PI(4,5)P_2_ and PI(3,4,5)P_3_ occupy a ring-shaped domain in close proximity to each other on the inner leaflet of the transition zone membrane.

**FIGURE 4:**
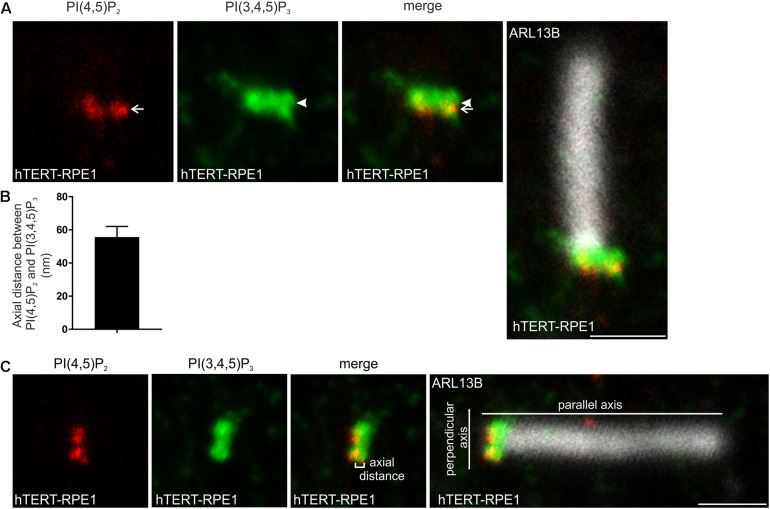
PI(4,5)P_2_ and PI(3,4,5)P_3_ localize to distinct subdomains within the transition zone. **(A)** Ciliated hTERT-RPE1 cells were immunostained with PI(4,5)P_2_ (red), PI(3,4,5)P_3_ (green) and ARL13B (grayscale) antibodies and imaged by STED microscopy (confocal resolution image of the ARL13B stained axoneme is shown). Arrow indicates PI(4,5)P_2_ transition zone signal and arrow head indicates PI(3,4,5)P_3_ transition zone signal, bar indicates 1 μm. **(B)** Graph shows the axial distance between the highest intensity point of the PI(4,5)P_2_ and PI(3,4,5)P_3_ signals parallel to the plane of the axoneme. Bars represent mean ± SEM, *n* = 3 independent experiments, 30 cilia imaged per experiment and all cilia with distinct PI(4,5)P_2_ and PI(3,4,5)P_3_ puncta measured. **(C)** Example image showing method for the axial distance measurements, bar indicates 1 μm.

Direct comparison of the transition zone distribution of PI(4,5)P_2_ and PI(3,4,5)P_3_ was performed in hTERT-RPE1 cells. Consistent with the maps of the individual PIs with TCTN1, the highest intensity point of the PI(4,5)P_2_ signal did not co-localize with PI(3,4,5)P_3_ (Figure 4A). PI(3,4,5)P_3_ was ∼50 nm more distal than PI(4,5)P_2_ (Figures 4A–C), consistent with an interpretation that each PI occupies a distinct subdomain of the transition zone membrane in the axial plane.

**FIGURE 5:**
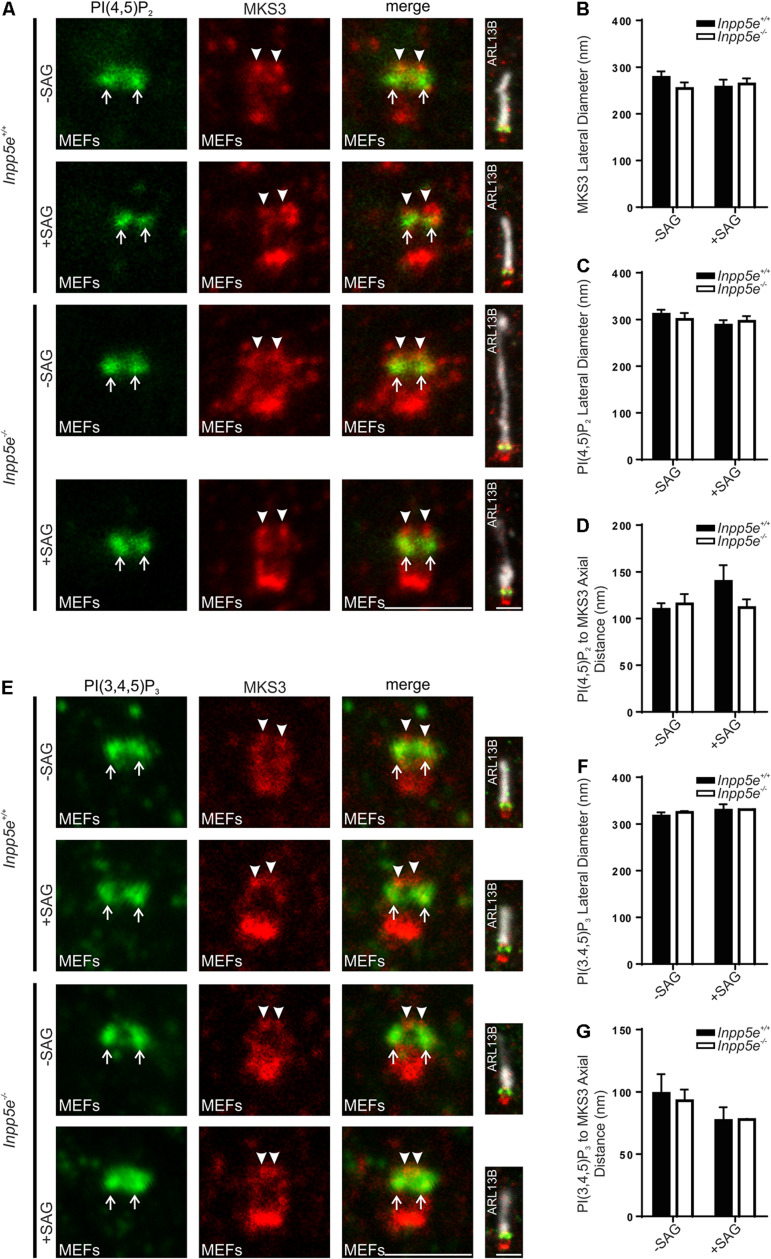
Transition zone phosphoinositide subdomain localization is not altered by stimulation or loss of a regulatory 5-phosphatase, INPP5E. **(A–G)** Ciliated *Inpp*5*e*^+ ⁣/ +^ and *Inpp5e*^–/–^ MEFs cells were treated + /- 100 nM SAG and immunostained with **(A)** PI(4,5)P_2_ or **(E)** PI(3,4,5)P_3_ (green), MKS3 (red) and ARL13B (grayscale) antibodies and imaged by STED microscopy (confocal resolution image of the ARL13B stained axoneme is shown). Right panels show merged image at lower magnification. Arrows indicate transition zone PI signals, arrow heads indicate MSK3 localization, bar indicates 1 μm. Graph shows the lateral diameter between the highest intensity points of the **(B)** MKS3, **(C)** PI(4,5)P_2_ or **(F)** PI(3,4,5)P_3_ puncta perpendicular to the plane of the axoneme. Bars represent mean ± SEM, *n* = 3 independent experiments, 30 cilia imaged per experiment and all cilia with two distinct PI or transition zone protein puncta measured. Graph shows the axial distance between the highest intensity point of the **(D)** PI(4,5)P_2_ or **(G)** PI(3,4,5)P_3_ signal and the MKS3 signal parallel to the plane of the axoneme. Bars represent mean ± SEM, *n* = 3 (*n* = 2 for SAG treated *Inpp5e*^–/–^ cells) independent experiments, 30 cilia imaged per experiment and all cilia with distinct PI or MKS3 puncta measured.

We have previously reported the levels (intensity) of transition zone PI(4,5)P_2_ and PI(3,4,5)P_3_ signals are changed upon INPP5E deletion or activation of Hedgehog signaling in response to the synthetic Smoothened agonist (SAG), as detected by confocal microscopy (Conduit et al., 2017; Dyson et al., 2017). We asked whether these stimuli also alter the domain organization of these transition zone phosphoinositides as assessed using superresolution microscopy. As STED microscopy rapidly bleaches fluorophores due to the nature of the superresolution mechanism and is not widely used for quantitative assessment of fluorescence intensity, this imaging modality was used here specifically to examine localization and not the fluorescence intensity of the PI signal. MKS3 was used as a marker to place the PIs in context. In both *Inpp*5*e*^+ ⁣/ +^ and *Inpp5e*^–/–^ MEFs, MKS3 exhibited two distinct puncta at the transition zone with a lateral diameter slightly less than 300 nm (Figures 5A,B). PI(4,5)P_2_ staining showed a positive domain with a lateral diameter wider than and >100 nm proximal to MKS3 (Figures 5A,C,D). This distribution was unchanged between *Inpp*5*e*^+ ⁣/ +^ and *Inpp5e*^–/–^ MEFs and following SAG stimulation (Figures 5A,C,D). In MEFs PI(3,4,5)P_3_ exhibited two puncta with a lateral diameter of approximately 300 nm and an axial distance slightly less than 100 nm proximal to MKS3 (Figures 5E,F,G). PI(3,4,5)P_3_ transition zone domain localization was not altered by deletion of *Inpp5e* or activation of Hedgehog signaling with SAG (Figures 5E,F,G). Interestingly the PI(4,5)P_2_, PI(3,4,5)P_3_ and MKS3 transition zone subdomains observed in MEFs are similar to but slightly condensed compared to the distributions defined in hTERT-RPE1 cells (see Figures 2, 3, 5), consistent with the known cell type-specific differences in cilia morphology (Yang et al., 2015).

### Activated AKT Localizes to the Transition Zone in Close Proximity to the PIs

**FIGURE 6:**
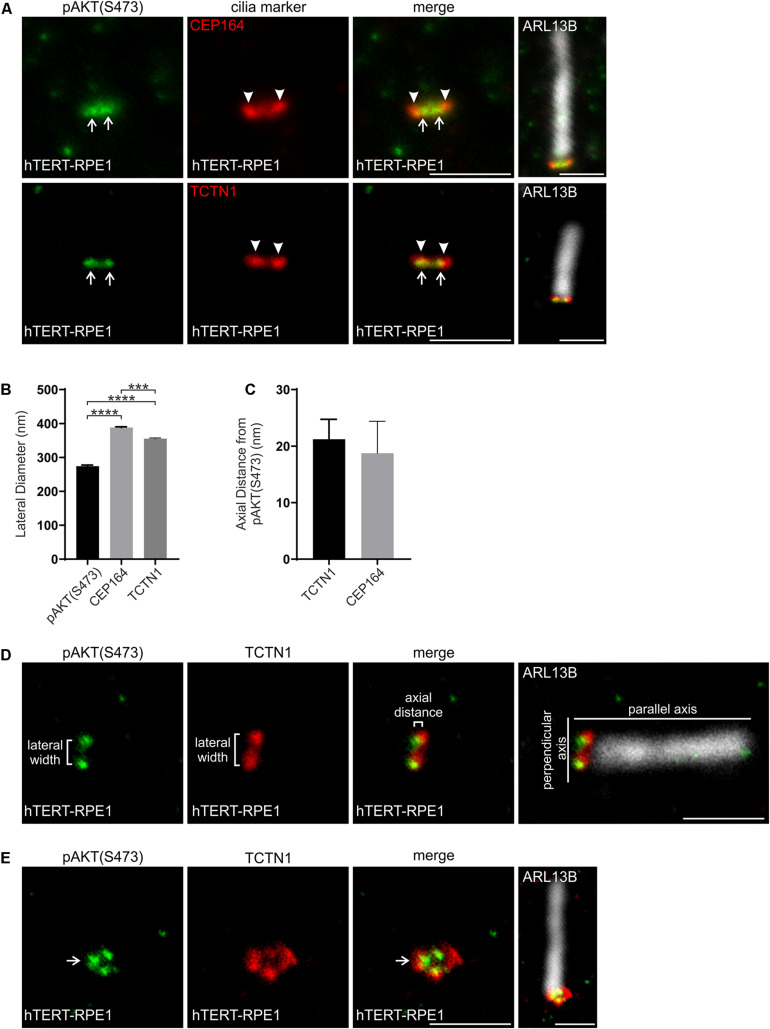
pAKT(S473) localizes in close proximity to the PIs at the transition zone. **(A)** Ciliated hTERT-RPE1 cells were immunostained with pAKT(S473) (green), CEP164 or TCTN1 (red) and ARL13B (grayscale) antibodies and imaged by STED microscopy (confocal resolution image of the ARL13B stained axoneme is shown). Right panels show merged image at lower magnification. Arrows indicate transition zone pAKT(S473) signal, arrow heads indicate CEP164 or TCTN1, bar indicates 1 μm. **(B)** Graph shows the lateral diameter between the highest intensity points of the pAKT(S473), CEP164 or TCTN1 puncta perpendicular to the plane of the axoneme. Bars represent mean ± SEM, *n* = 3 independent experiments, ≥30 cilia imaged per experiment and all cilia with two distinct pAKT(S473), CEP164 or TCTN1 puncta measured, statistical significance was determined using one-way ANOVA (*p* < 0.0001) followed by Tukey’s post hoc test, ***p < 0.001, ****p < 0.0001. **(C)** Graph shows the axial distance between the highest intensity point of the pAKT(S473) signal and CEP164 or TCTN1 parallel to the plane of the axoneme. Bars represent mean ± SEM, *n* = 3 independent experiments, ≥ 30 cilia imaged per experiment and all cilia with distinct pAKT(S473), CEP164 or TCTN1 puncta measured, statistical significance was determined using Student’s t-test (*p* = 0.7284). **(D)** Representative image showing the method used for the lateral diameter and axial distance measurements, bar indicates 1 μm. **(E)** Ciliated hTERT-RPE1 cells were immunostained with pAKT(S473) (green), TCTN1 (red) and ARL13B (grayscale) antibodies and imaged by STED microscopy (confocal resolution image of the ARL13B stained axoneme is shown). Arrow indicates ring shaped transition zone pAKT(S473) morphology, bar indicates 1 μm.

The serine/threonine kinase AKT is a key PI(3,4,5)P_3_ effector which is recruited to membranes via its PI(3,4,5)P_3_ and PI(3,4)P_2_ binding PH domain. This interaction brings AKT into close proximity to its activating kinases PDK1 and mTORC2 which phosphorylate AKT at T308 and S473 respectively (Frech et al., 1997; Stephens et al., 1998; Sarbassov et al., 2005; Heras-Martínez et al., 2019). Phosphorylated AKT (both pAKT(S473) and pAKT(T308)) has been identified at the cilia base and is associated with destabilization of the cilium (Zhu et al., 2009; Schneider et al., 2010; Higginbotham et al., 2013; Suizu et al., 2016; Conduit et al., 2017). However, the pAKT sub-ciliary localization is uncharacterized. We assessed whether the ciliary base pAKT(S473) corresponded to the transition zone and compared it to the map developed here in hTERT-RPE1 cells using superresolution microscopy. pAKT(S473) was observed as two distinct puncta at the transition zone consistent with a ring-shape domain with a lateral diameter of ∼270 nm, significantly narrower than the TCTN1 and CEP164 domains (Figures 6A,B,D,E). The pAKT(S473) ring was located within the PI(3,4,5)P_3_ and PI(4,5)P_2_ rings, which have a lateral diameter of ∼300 nm, consistent with a model in which cytosolic AKT binds membrane-associated PIs at the transition zone. In the axial plane, the peak intensity of the pAKT(S473) puncta was in very close proximity (∼20 nm, beyond the limit of STED resolution) to the peak intensity of the TCTN1 and CEP164 signals (Figures 6A,C,D), indicating localization to the most proximal region of the transition zone, similar to PI(4,5)P_2_. No change in pAKT(S473) localization was observed following IGF-1 stimulation (2 min-2 h) of hTERT-RPE1 cells (Supplementary Figure 2, representative image of 5 min stimulation shown).

### INPP5E Is Concentrated at the Cilia Transition Zone

**FIGURE 7:**
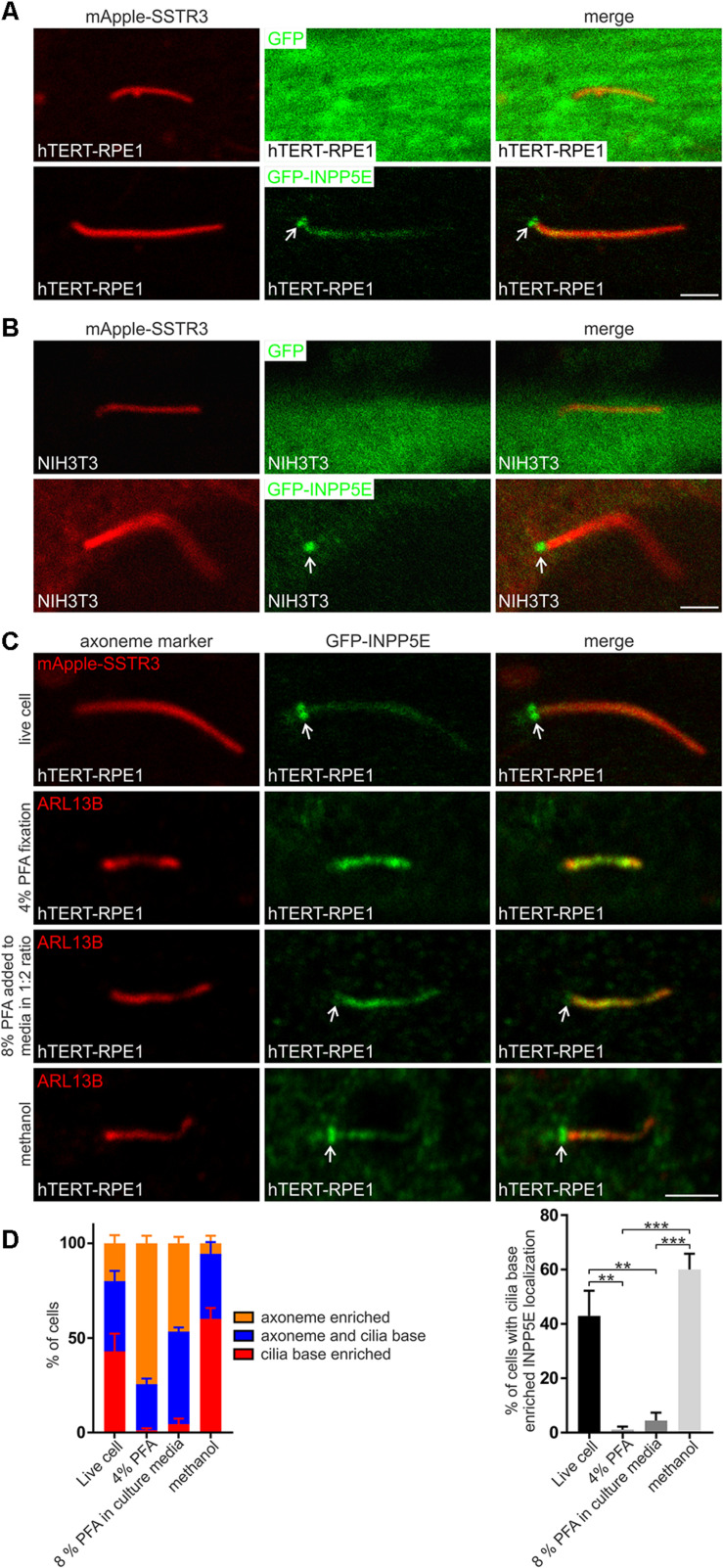
INPP5E localizes to the transition zone. **(A)** hTERT-RPE1 or **(B)** NIH3T3 cells were transfected with mApple-SSTR3 and GFP or GFP-INPP5E, serum starved and imaged live by confocal microscopy. Arrow indicates transition zone INPP5E localization, bar indicates 2 μm. **(C)** hTERT-RPE1 cells were transfected with GFP-INPP5E ± mApple-SSTR3, serum starved and imaged live or immunostained with GFP (green) and ARL13B (red) antibodies using 4% PFA, 8% PFA added directly to the culture media in a 1:2 ratio or –20°C methanol fixation and imaged by confocal microscopy. Arrow indicates transition zone INPP5E localization, bar indicates 2 μm. **(D)** Graphs show the percentage of cells exhibiting exclusive axoneme, exclusive transition zone or axoneme and transition zone INPP5E localization. Bars represent mean ± SEM, *n* = 3 independent experiments, 30 cilia imaged per experiment, statistical significance was determined by one-way ANOVA (*p* = 0.0002) followed by Tukey’s post hoc test **p < 0.01, ***p < 0.001.

INPP5E is a significant regulator of transition zone PIs, however, all reports to date have shown endogenous and recombinant INPP5E localize to the ciliary axoneme away from its substrates (Bielas et al., 2009; Jacoby et al., 2009; Humbert et al., 2012; Plotnikova et al., 2015; Conduit et al., 2017; Dyson et al., 2017; Qiu et al., 2021). We previously showed that in a minority of cells INPP5E also partially co-localized with TCTN1 at the transition zone (Dyson et al., 2017). Here we investigated green fluorescent protein (GFP)-INPP5E localization in hTERT-RPE1 cells by live cell imaging to remove any possible fixation, permeabilization and/or staining artifacts that could affect the INPP5E distribution. Cells also co-expressed mApple-SSTR3 as an axoneme membrane marker. GFP-INPP5E was observed in the axoneme but was notably concentrated at the cilia base in a distribution characteristic of the transition zone (Figure 7A), suggesting that in live cells the 5-phosphatase may predominantly localize to the transition zone in the vicinity of its PI substrates. The cilia base concentration of INPP5E was confirmed in a second cell line via live cell imaging of ciliated NIH3T3 cells expressing GFP-INPP5E and mApple-SSTR3 (Figure 7B). GFP exhibited a diffuse cytosolic pattern in NIH3T3 cells (Figure 7B). The difference in INPP5E localization observed here in live cells compared to the axoneme-specific distribution reported previously in fixed cells, including hTERT-RPE1 cells, suggests fixation and staining protocols may affect the apparent localization. We directly compared GFP-INPP5E localization in live hTERT-RPE1 cells with cells fixed using a standard 4% PFA method followed by 0.1% Triton X-100 permeabilization. In the majority of live cells GFP-INPP5E was concentrated at the cilia base with lower levels in the axoneme, whereas 4% PFA fixation induced an almost exclusive axoneme distribution (Figures 7C,D), similar to that reported previously (Bielas et al., 2009; Jacoby et al., 2009; Humbert et al., 2012; Plotnikova et al., 2015; Conduit et al., 2017; Dyson et al., 2017; Qiu et al., 2021). In the small proportion of PFA (4%) fixed cells exhibiting cilia-base INPP5E signals, INPP5E co-localized with TCTN1 at the transition zone (Supplementary Figure 3A). Two additional fixation methods, a 1:2 dilution of 8% PFA into the culture media or 100% −20°C methanol, were also used to determine whether the transition zone INPP5E distribution observed in live cells could be recapitulated in fixed cells. A 1:2 dilution of 8% PFA into the culture media partially retained the cilia base GFP-INPP5E signal (Figures 7C,D). Interestingly, fixation using 100% −20°C methanol retained the GFP-INPP5E concentration at the cilia base, similar to live cells. We conclude INPP5E is concentrated at the cilia base, consistent with the transition zone as we observed co-localization with TCTN1 in a small subset of PFA fixed cells. As 100% −20°C methanol fixation most closely replicated the live cell INPP5E distribution, we used this approach for subsequent fixed cell INPP5E localization studies.

Upon serum stimulation, INPP5E exits the cilia axoneme in a process that contributes to cilia disassembly (Phua et al., 2017). We examined whether the transition zone INPP5E localization in live cells was also regulated by cilia disassembly stimuli. hTERT-RPE1 cells expressing GFP-INPP5E were imaged live with or without 5 h 10% FBS stimulation. Serum stimulation did not alter GFP-INPP5E localization. In both starved and stimulated cells, low level axoneme INPP5E was observed with significant 5-phosphatase concentration at the transition zone (Supplementary Figure 3B). GFP-INPP5E localization was also assessed at various timepoints during the 5 h serum stimulation time course and no changes in its localization were observed (not shown). Therefore, in live cells INPP5E constitutively concentrates at the transition zone in close proximity to its PI substrates and its distribution is unchanged by stimulation that at over a longer time course causes cilia disassembly.

**FIGURE 8:**
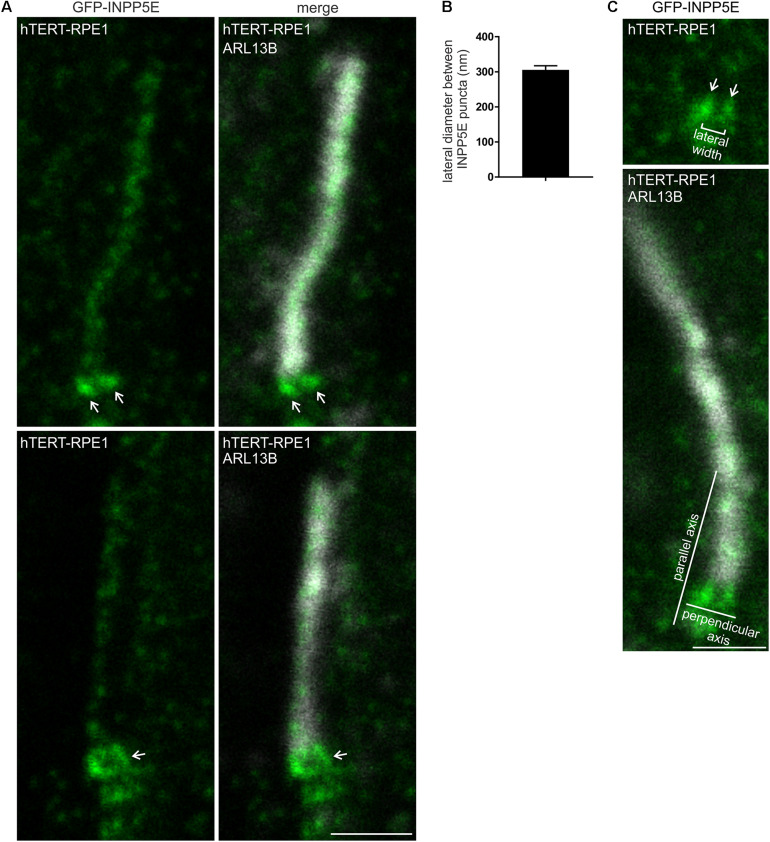
INPP5E exhibits a ring-shaped localization at the transition zone. **(A)** hTERT-RPE1 cells were transfected with GFP-INPP5E, serum starved and immunostained with GFP (green) and ARL13B (grayscale) antibodies and imaged by STED microscopy (confocal resolution image of the ARL13B stained axoneme is shown). Arrows indicate transition zone INPP5E localization, bar indicates 1 μm. **(B)** Graph shows the lateral diameter between the highest intensity points of the INPP5E transition zone protein puncta perpendicular to the plane of the axoneme. Bars represent mean ± SEM, *n* = 3 independent experiments, 30 cilia imaged per experiment and all cilia with two distinct INPP5E puncta measured. **(C)** Example image showing method for the lateral diameter and axial distance measurements, bar indicates 1 μm.

To examine the transition zone INPP5E localization in more detail, GFP-INPP5E was assessed by STED microscopy in methanol fixed hTERT-RPE1 cells. Some GFP-INPP5E was observed in the axoneme, co-localizing with ARL13B (Figure 8A). However, the major GFP-INPP5E signal was concentrated at the cilia base with two distinct signal intensity peaks (Figure 8A), similar to PI(4,5)P_2_, PI(3,4,5)P_3_ and some transition zone proteins described above (see Figures 2, 3). In cells where the cilia were at an angle to the plane of imaging, a ring-shaped GFP-INPP5E signal was observed (Figure 8A). The lateral diameter between the highest intensity point of each INPP5E punctum in cilia with two distinct intensity peaks was ∼300 nm (Figures 8B,C), comparable to the lateral diameter of the transition zone membrane component PIs (Figures 2, 3). As INPP5E is membrane associated via its CAAX motif and we have shown co-localization between INPP5E and TCTN1 (see Supplementary Figure 3A)(Dyson et al., 2017), we propose INPP5E is associated with the inner leaflet of the cilia transition zone membrane in proximity to its PIs substrates.

## Discussion

**FIGURE 9:**
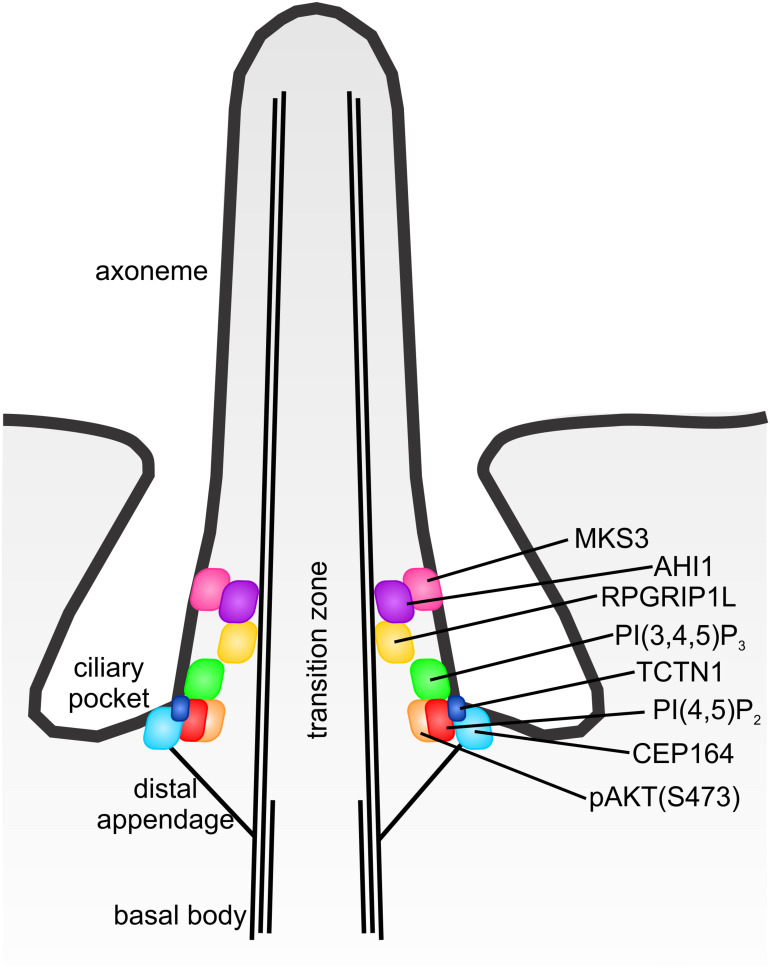
Model of transition zone phosphoinositide localization. Model depicting the superresolution localization of PI(4,5)P_2_, PI(3,4,5)P_3_ and pAKT(S473) in the context of the transition zone protein and distal appendage components.

In this study we report the molecular organization of the PIs PI(4,5)P_2_ and PI(3,4,5)P_3_ and their effector pAKT(S473) at the cilia transition zone in the landscape of transition zone proteins and distal appendages (Figure 9). Transition zone barrier function is essential for cilia assembly and signaling (Chih et al., 2011; Garcia-Gonzalo et al., 2011; Sang et al., 2011; Gonçalves and Pelletier, 2017). Mutations in transition zone components disrupt barrier function and are a major cause of ciliopathy syndromes (Gonçalves and Pelletier, 2017). In addition, deletion of the Joubert syndrome gene *Inpp5e* disrupts transition zone PI turnover associated with mislocalization of transition zone proteins and loss of barrier retention function (Dyson et al., 2017). Interestingly, we provide evidence that INPP5E is enriched at the transition zone, close to its PI substrates. These findings add to the growing body of evidence that Joubert syndrome is caused by mutations in transition zone proteins (Chih et al., 2011; Garcia-Gonzalo et al., 2011; Huang et al., 2011; Sang et al., 2011; Damerla et al., 2015; Lambacher et al., 2016; Gonçalves and Pelletier, 2017).

PI(4,5)P_2_ and PI(3,4,5)P_3_ have been detected at cilia in multiple studies, however, various disparate localizations for the PIs within the cilia subdomains including at the transition zone, axoneme and cilia base have been reported (Chavez et al., 2015; Garcia-Gonzalo et al., 2015; Jensen et al., 2015; Park et al., 2015; Conduit et al., 2017; Dyson et al., 2017; Prosseda et al., 2017; Conduit and Vanhaesebroeck, 2020). PI binding biosensors have been widely used for PI detection but are limited as they may sequester lipid in some contexts and may not always accurately reflect PI localization when expressed at high levels (Hammond and Balla, 2015; Tsuji et al., 2019). PI specific antibodies are increasingly used for PI detection but may be sensitive to membrane permeabilization and fixation conditions (Hammond et al., 2009). In an attempt to resolve the reported disparate PI distributions we compared the PI(4,5)P_2_ and PI(3,4,5)P_3_ localization obtained using multiple fixation, permeabilization and staining methods with validated PI antibodies, revealing PI(4,5)P_2_ and PI(3,4,5)P_3_ exhibit ring like domains at the inner leaflet of the most proximal region of the transition zone membrane.

The PI rings occupy very similar domains to TCTN1 in the lateral and axial planes, although PI(3,4,5)P_3_ is more distal to PI(4,5)P_2_. The PIs are wider and more proximal than MKS3, AHI1 and RPGRIP1L. CEP164 marks the distal appendages and demarks the proximal boundary of the transition zone (Yang et al., 2015). PI(4,5)P_2_ was the proximal most component we examined and localized very close to CEP164 (<50 nm, the limit of STED resolution) in the axial plane but exhibited a significantly narrower lateral diameter (Figure 9). MKS3 is a transmembrane protein, while AHI1 and RPGRIP1l contain putative PI binding domains (Garcia-Gonzalo and Reiter, 2012; Remans et al., 2014), suggesting these proteins are membrane associated. Surprisingly these transition zone components exhibit a lateral width ∼100 nm less than the membrane associated PI ring. However, consistent with a previous study (Yang et al., 2015), we observed two distinct layers of transition zone components in the axial plane (Figure 9). TCTN1, PI(4,5)P_2_ and PI(3,4,5)P_3_ are located in the most proximal region of the transition zone, very close to the distal appendages, whereas MKS3, AHI1 and RPGRIP1L form the distal group. This suggests the ciliary membrane may narrow at the level of the distal group enabling components at both axial levels to contact the membrane (Figure 9).

It is difficult to precisely predict where the PI decorated transition zone membrane sits in relation to the CEP164 stained distal appendages. Previous studies have not shed light on this interesting question as immunoelectron microscopy experiments showing CEP164 localizes to the distal appendages were performed in non-ciliated U2OS cells (Graser et al., 2007). Subsequent assessments by superresolution microscopy overlayed an averaged representative fluorescent CEP164 stained distal appendage image with an electron micrograph of a different cell and did not co-stain the same cell with CEP164 antibodies and a fluorescent membrane marker (Yang et al., 2015, 2018). The localization of PI(4,5)P_2_ relative to CEP164 observed here (similar axial plane but narrower lateral diameter) appears to indicate that the PI(4,5)P_2_ positive membrane is within the ring created by the distal appendages consistent with the proximal end of the transition zone rather than at the distal tips of the distal appendages (Figure 9).

PI(4,5)P_2_ and PI(3,4,5)P_3_ localize to distinct subdomains of the transition zone membrane, with PI(4,5)P_2_ more proximal to TCTN1 and PI(3,4,5)P_3_ more distal (Figure 9). The reason for this spatial separation is unknown but may be important for the recruitment of distinct effectors to different regions of the transition zone. The PI 5-phosphatase INPP5E which contains a C-terminal membrane targeting CAAX motif also showed a lateral diameter of ∼300 nm, similar to the PIs, suggesting it also localizes to the inner leaflet of the transition zone membrane in close proximity to its substrates. The PI(3,4,5)P_3_ effector pAKT(S473) localized to the proximal plane of the transition zone with the PIs but exhibited a slightly smaller lateral diameter than the PIs (Figure 9). Surprisingly, the pAKT(S473) domain was closer to PI(4,5)P_2_ than its binding partner PI(3,4,5)P_3_ in the axial plane. Active AKT has been identified in the cytosol away from PI(3,4,5)P_3_ containing membranes (Kunkel et al., 2005), suggesting that perhaps following transient PI(3,4,5)P_3_ binding and phosphorylation, pAKT(S473) accumulates at a distinct site at the transition zone near PI(4,5)P_2_ to exert its function, however, this prediction will need to be further investigated.

Stimulation of cells with growth factors or deletion of *Inpp5e* has previously been shown to alter the levels of cilia PIs and their regulators (Conduit et al., 2017; Dyson et al., 2017; Phua et al., 2017). Here we observed that the same stimuli did not alter PI(4,5)P_2_, PI(3,4,5)P_3_ or pAKT(S473) transition zone localization, indicating that although these components turn over and the levels are dynamic, their domain organization is constant. We hypothesize INPP5E is critical for regulating the levels of the PIs at the transition zone but factors other than the 5-phosphatase are responsible for restricting PIs to their specific subdomains. Lipid diffusion barriers have long been thought to exist at the transition zone and contribute to the asymmetric lipid composition of the cilia and plasma membranes (Hu and Nelson, 2011; Trimble and Grinstein, 2015; Garcia et al., 2018). Although the nature and mechanism of action of potential lipid diffusion barrier(s) remains elusive, it is possible they contribute to control of the PI domain localizations within the transition zone. It is also possible that the lipid domains are established by the localized production of these species by yet to be identified transition zone localized PI-kinases. Alternatively, PI binding scaffold proteins that generate local PI-enriched membrane domains have recently been identified at other sites in the cell and may play a role in establishing the transition zone PI domains. It is interesting to speculate that IFITM3 may serve this role for PI(3,4,5)P_3_ as it has recently been shown to form PI(3,4,5)P_3_ rich membrane regions in B cells and localize to epithelial cell motile cilia (Bailey et al., 2014; Lee et al., 2020).

The use of STED microscopy has greatly improved the resolution limit of imaging cilia compared to confocal microscopy allowing the mapping of PIs and effectors in the context of the transition zone proteins. However, this technique is still limited by optical resolution and indirect IF microscopy experimental constraints. The requirement for primary and secondary antibodies for protein or PI detection adds ∼16 nm to the epitope (Yang et al., 2015). This level of uncertainty is unlikely to have a major effect here as STED microscopy is limited to an optical resolution of ∼50 nm (Yang et al., 2015). Most of the dimensions measured here are greater than 50 nm, however, the PIs and pAKT(S473) were located in very close proximity to TCTN1 and CEP164 in the axial plane. Therefore, we do not draw conclusions from measurements of <50 nm other than to indicate that two components are in very close proximity.

The transition zone molecular map we have built here provides an ideal basis to formulate testable hypotheses for how the PIs regulate transition zone function. Many of the transition zone proteins including RPGRIP1L and AHI1 contain putative PI binding C2 and B9 domains (Garcia-Gonzalo and Reiter, 2012; Remans et al., 2014). This raises the possibility that PI binding tethers the proteins at the transition zone. However, the transition zone PI map (Figure 9) reveals these PI binding domain proteins occupy a distinct axial plane compared to the PI(4,5)P_2_ and PI(3,4,5)P_3_, making this unlikely. Furthermore, the RPGRIP1L C2 domains exhibit a negative charge which means PI binding is improbable (Remans et al., 2014). PI(3,4,5)P_3_ and PI(4,5)P_2_ are in closest proximity to TCTN1, but an interaction between TCTN1 and the PIs also seems unlikely given this transition zone protein contains a signal peptide and it has been suggested to associate with the outer leaflet of the transition zone membrane (Garcia-Gonzalo et al., 2011), whereas the PIs may decorate the inner leaflet. Collectively, these data suggest that if the PIs tether transition zone components, they must bind to alternative transition zone complex members not investigated here. Alternatively, it is possible that the PIs may act via an indirect mechanism to regulate barrier function mediated by PI effector proteins such as AKT which localizes to the proximal transition zone subdomain close to the PIs.

In summary, here we have constructed a superresolution map of PI(3,4,5)P_3_ and PI(4,5)P_2_ at the transition zone in the context of the transition zone proteins and distal appendages, a key PI regulator INPP5E and the PI effector protein pAKT(S473). We identify that the PIs and their regulator/effector localize to a proximal transition zone subdomain with TCTN1, close to the distal appendages, but each component exhibits a distinct distribution within this compartment. This map will provide an ideal guide to base future studies into the long-standing question of how the transition zone functions as a molecular gate with important implications for our understanding of the molecular basis of ciliopathy syndromes.

## Materials and Methods

### Antibodies and Reagents

Antibodies used were: ARL13B (IF: 1:200, N2956/66) and GFP (IF: 1:500, ab290) from Abcam (Cambridge, MA, United States). PI(3,4,5)P_3_ (IF: 1:100, Z-P345B), PI(4,5)P_2_ (IF:1:500, Z-P045) and PI(4)P (IF:1:500, Z-P004) from Echelon (Santa Clara, CA, United States). CEP164 (IF: 1:500, NBP1-81445) was from Novus Biologicals (Centennial, CO, United States). AHI1 (IF:1:100, 22045-1-AP), MKS3 (IF: 1:200, 13975-1-AP) and TCTN1 (IF: 1:100, 15004-1-AP) from Proteintech (Rosemont, IL, United States). RPGRIP1L (IF 1:100, HPA039405) was from Sigma-Aldrich (St. Louis, MO, United States). pAKT(S473) (IF:1:500, 4051) was from Cell Signaling Technology (Danvers, MA, United States). V5 (IF 1:1000, R960-25) was from Life Technologies (Carlsbad, CA, United States). Alexa-Fluor-488/594/647-conjugated mouse and rabbit secondary antibodies (IF 1:600) were from Life Technologies. Star 580-conjugated rabbit secondary antibodies were from Abberior (Göttingen, Germany). pEGFP-C2 (GFP vector) was from Clonetech, pEGFP-C2-INPP5E (GFP-INPP5E) was described previously (Kong et al., 2000). pLenti6.2/V5-DEST-INPP5E (V5-INPP5E) was described previously (Plotnikova et al., 2015). mApple-SSTR3-N-17 was a gift from Michael Davidson (Addgene plasmid # 54949)^1^. All other reagents were from Sigma-Aldrich unless specified.

### *Inpp5e* Knockout Mice

Procedures using mice were approved by the Monash Animal Research platform animal ethics committee, Monash University. All mouse strains used were on the C57BL/6 background. *Inpp5e*^–/–^ (*Inpp5e*^*tm1.1Cmit*^) embryos were generated and described previously by crossing *Inpp*5*e*^+ ⁣/−^ mice (Dyson et al., 2017).

### Cell Culture and Live Cell Imaging

hTERT-RPE1 cells were purchased from ATCC and cultured in DMEM-F12 with 10% fetal calf serum and 0.01 mg/ml hygromycin B. Primary cilia were induced by 48 h serum starvation. NIH3T3 cells were purchased from Sigma-Aldrich. NIH3T3 cells were cultured in DMEM with 10% neonatal calf serum (Life Technologies), 0.1% (w/v) streptomycin, 100 units/ml penicillin and 2 mM L-glutamine (Sigma-Aldrich, 59202C). Cilia assembly was induced by 48 h serum starvation. For growth factor stimulation, ciliated cells were stimulated with 10 nM IGF-1 for 0, 2, 5, 15, 60 or 120 min or 10% FBS for 0–5 h followed by fixation and staining or live cell imaging. hTERT-RPE1 and NIH3T3 cells were transfected using Lipofectamine 2000 (Life Technologies) according to manufactures specifications.

For live cell imaging, cells were seeded into Flurodishes (Ibidi, Planegg, Germany) transfected and serum starved. The media was replaced with phenol red free DMEM:F12 or DMEM containing 1 μM Hoechst Dye (Sigma-Aldrich 33342) at least 10 min prior to imaging. Live cell imaging was performed at 37°C. See microscopy section for details of imaging techniques.

MEFs were harvested from E12.5 embryos. Pregnant female mice were humanely sacrificed and the embryos harvested, decapitated and eviscerated. The tissue was washed in sterile PBS and minced with a scalpel blade. Cells were disassociated by incubation in 0.25% Trypsin, 0.02% EDTA at 37°C, 5% CO_2_ for 5 min. The cell suspension was plated in DMEM containing 10% FBS with 0.1% (w/v) streptomycin, 100 units/ml penicillin and 2 mM L-glutamine (Sigma-Aldrich, 59202C) and incubated at 37°C, 5% CO_2_. Primary cilia assembly was induced by 48 h serum starvation.

### Immunofluorescence

Phosphoinositide staining was performed using the Golgi protocol described by Hammond et al. (2009), or the staining protocols described by Chavez et al. (2015), Yang et al. (2015), or Garcia-Gonzalo et al. (2011) as indicated in the figures and figure legends. Alternatively, for PI and pAKT(S473) IF cells were fixed with 4% PFA for 20 min, washed in PBS, permeabilized with 100% methanol at −20°C for 5 min, washed in PBS and blocked with PBS containing 1 % (w/v) BSA for 30 min. Primary antibodies were diluted in block and incubated for 1 h at room temperature, following three washes, secondary antibodies were diluted in block and incubated for 45 min at room temperature. Cells were then washed three times and mounted with Fluromount G (Electron Microscopy Sciences, Hatfield, PA, United States).

IF to detect INPP5E at the cilium was performed by fixing cells with either 4% PFA for 20 min or 8% PFA diluted 1:2 directly into the culture media for 20 min or an optimized methanol-based fixation technique. Cells were then washed and permeabilized with 0.1% (v/v) triton X-100 in PBS for 90 s followed by washing Samples were blocked with 1% (w/v) BSA for 30 min. Primary antibodies were diluted in block and incubated for 1 h at room temperature. Following three washes, secondary antibodies were diluted in block and incubated for 45 min. Finally, cells were washed three times with PBS and mounted using Fluormount G.

Optimized methanol-based staining for INPP5E detection was performed using 100% methanol to fix cells for 10 min at −20°C. Cells were then washed three times with room temperature PBS and blocked with 1% (w/v) BSA in PBS for 30 min at room temperature. Primary antibodies (diluted in 1% (w/v) BSA, antibody dilutions described above) were applied to cells for 1 h at room temperature followed by three PBS washes. Secondary antibodies (antibody dilutions described above) were diluted in 1% (w/v) BSA and incubated with the cells for 45 min at room temperature. Cells were then washed three times with PBS and mounted onto glass slides using Fluormount G.

### Microscopy

Microscopy was performed at Monash Micro Imaging, Monash University (Australia). Confocal microscopy was performed using a Leica TCS SP8 microscope with a 63x HC PL APO CS2 (11506350) 1.4 NA oil objective lens, HyD detector and Leica LAS X acquisition software. Confocal and STED imaging were performed using an Expert line STED (Abberior Instruments GmbH, Göttingen, Germany) microscope, based on an Olympus IX83 body with a ×100 oil/1.4NA (UPlanSApo,0.17 mm WD) objective lens and 3 Channel detector unit (APD’s), pulsed lasers for 485, 561, 640 nm excitation, with 1W STED lasers at 595 and 775 nm and Imspector acquisition software. Images were obtained using 20 nm sized pixels.

Image J software (National Institutes of Health, Rockville, MD, United States) was used for image processing and was limited to alterations of brightness, subjected to the entire image.

### Image Analysis

Image analysis was performed using Image J software. For lateral diameter and axial distance measurements, the parallel axis was defined using the ARL13B stained axoneme signal and the perpendicular axis defined as 90° to the parallel axis (Figures 3E, 4E). Lateral diameter measurements were performed for PI(3,4,5)P_3_, PI(4,5)P_2_, pAKT(S473), INPP5E, transition zone proteins and CEP164 from all images exhibiting two distinct signal intensity peaks. The lateral diameter was the distance in the perpendicular plane between the highest intensity point of each signal intensity peak. The axial distance was measured in all images with distinct PI(3,4,5)P_3_ or PI(4,5)P_2_, pAKT(S473) and transition zone protein or CEP164 signals. The axial distance was the distance in the parallel plane between the highest intensity points of the PI and the transition zone protein signals.

### Statistical Analysis

Statistical analysis was performed using GraphPad Prism 7 (San Diego, CA, United States). All graphs represent mean ± SEM. Differences were considered statistically significant when *p* < 0.05. *p*-values were calculated using either two tailed unpaired Student’s t tests with or without Welch’s correction for unequal variance as appropriate (difference in sample variance assessed by the F test), or one-way ANOVAs followed by Tukey’s post hoc test (difference in sample variance assessed by the Brown-Forsythe test) as indicated in the figure legend. The number of independent experiments is listed in the figure legends.

## Data Availability Statement

The raw data supporting the conclusions of this article will be made available by the authors, without undue reservation.

## Ethics Statement

The animal study was reviewed and approved by Monash Animal Research platform animal ethics committee, Monash University.

## Author Contributions

SC and CM conceived the project, designed and coordinated experiments, and wrote the manuscript. SC, ED, AF, and VO performed experiments and microscopy. SC, ED, and AF analyzed and interpreted data. All authors contributed to drafting the manuscript and approved the submitted version.

## Conflict of Interest

The authors declare that the research was conducted in the absence of any commercial or financial relationships that could be construed as a potential conflict of interest.

## Footnotes

1 http://n2t.net/addgene:54949; RRID:Addgene_54949.

## Abbreviations

GFP: green fluorescent protein
IF: immunofluorescence
IGF-1: insulin-like growth factor-1
MEFs: mouse embryonic fibroblasts
PFA: paraformaldehyde
PH: pleckstrin homology
PI: phosphoinositides
PI(3,4)P_2_: phosphatidylinositol 3, 4-bisphosphate
PI(3,4,5)P_3_: phosphatidylinositol 3, 4, 5-trisphosphate
PI(4)P: phosphatidylinositol 4 phosphate
PI(4,5)P_2_: phosphatidylinositol 4, 5-bisphosphate
PI3K: phosphoinositide 3-kinases
RPE: retinal pigment epithelial
SAG: Smoothened agonist
STED: stimulated emission depletion.
