# Supplementary material for: Superresolution Microscopy Reveals Distinct Phosphoinositide Subdomains Within the Cilia Transition Zone
Source: Front Cell Dev Biol. 2021 Apr 30;9:634649. doi: 10.3389/fcell.2021.634649 (PMC8120242; doi:10.3389/fcell.2021.634649)
Supplement: Supplementary file 1 [file Data_Sheet_1.PDF]

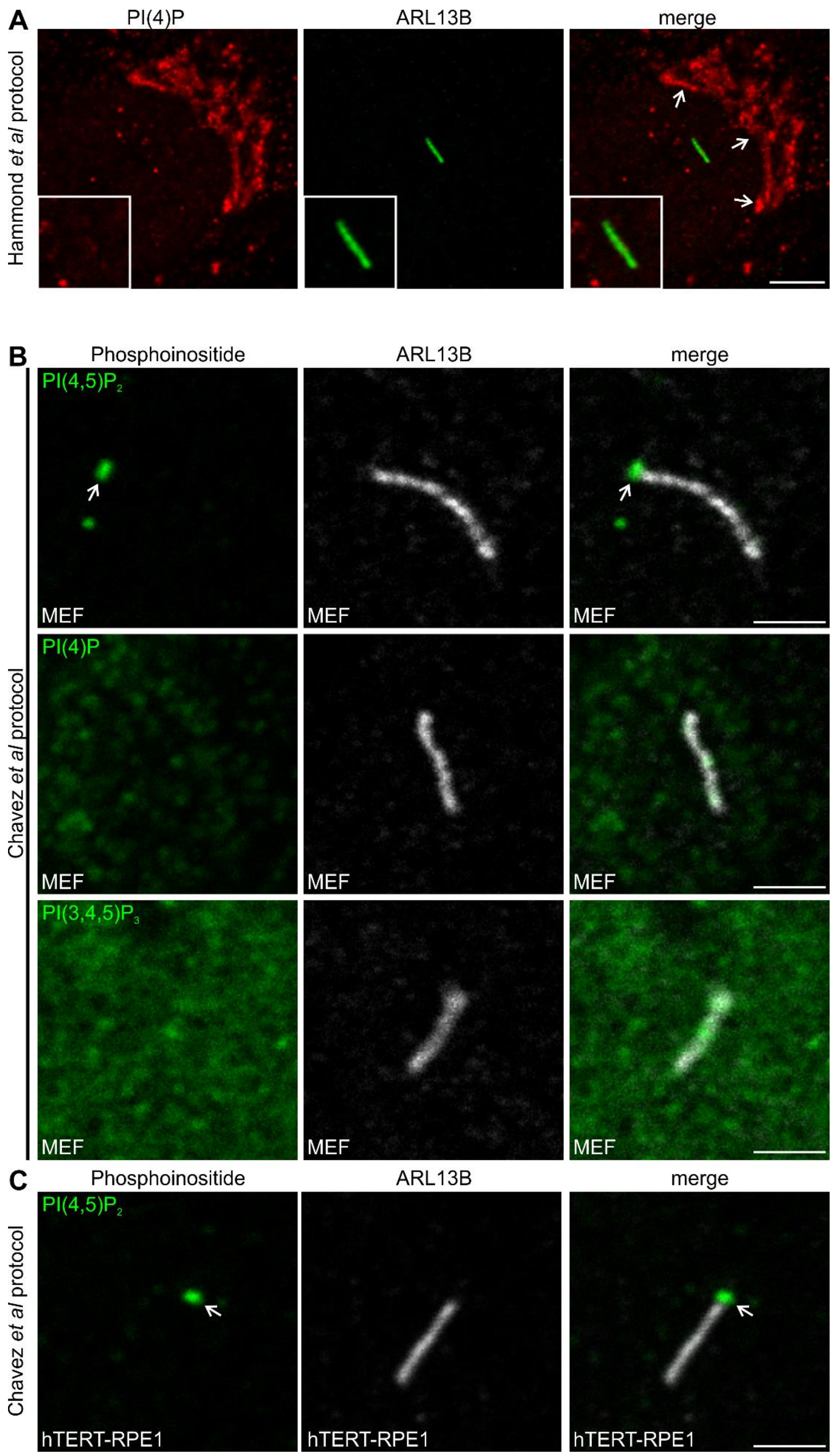


**Figure S1: Optimized staining conditions for detection of ciliary phosphoinositides**
**(A)** Ciliated hTERT-RPE1 cells were immunostained with PI(4)P (red) and ARL13B (green) antibodies using the Hammond et al (2009) Golgi protocol and imaged by confocal

microscopy, arrows indicate Golgi PI(4)P immunostaining, bar indicates 5 μm. **(B)** Ciliated wild type MEFs were immunostained with PI(4,5)$P_2$, PI(4)P or PI(3,4,5)$P_3$ (green) and ARL13B (grayscale) antibodies using the Chavez et al (2015) protocol and imaged by confocal microscopy, arrows indicate transition zone PI(4,5)$P_2$ signals, bar indicates 2 μm. **(C)** Ciliated hTERT-RPE1 cells were immunostained with PI(4,5)$P_2$ (green) and ARL13B (grayscale) antibodies using the Chavez et al (2015) protocol and imaged by confocal microscopy, arrows indicate transition zone PI(4,5)$P_2$ signals, bar indicates 2 μm.

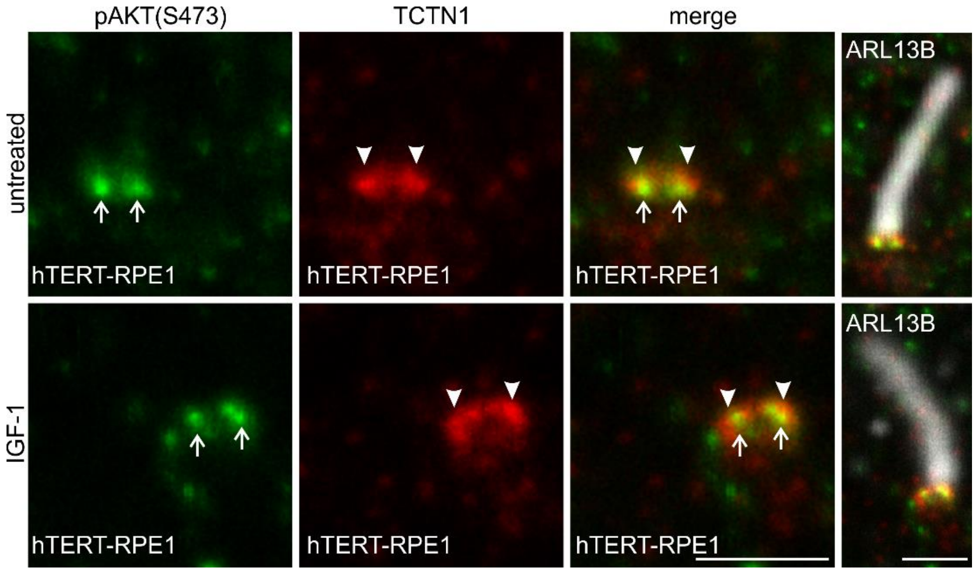


**Figure S2: IGF-1 stimulation does not modulate transition zone pAKT(S473) localization**

Ciliated hTERT-RPE1 cells were treated +/- 10 nM IGF-1 for 5 mins, immunostained with pAKT(S473) (green), TCTN1 (red) and ARL13B (grayscale) antibodies and imaged by STED microscopy (confocal resolution image of the ARL13B stained axoneme is shown). Right panels show merged image at lower magnification. Arrows indicate transition zone pAKT(S473) signal, arrow heads indicate TCTN1 signal, bar indicates 1 μm.

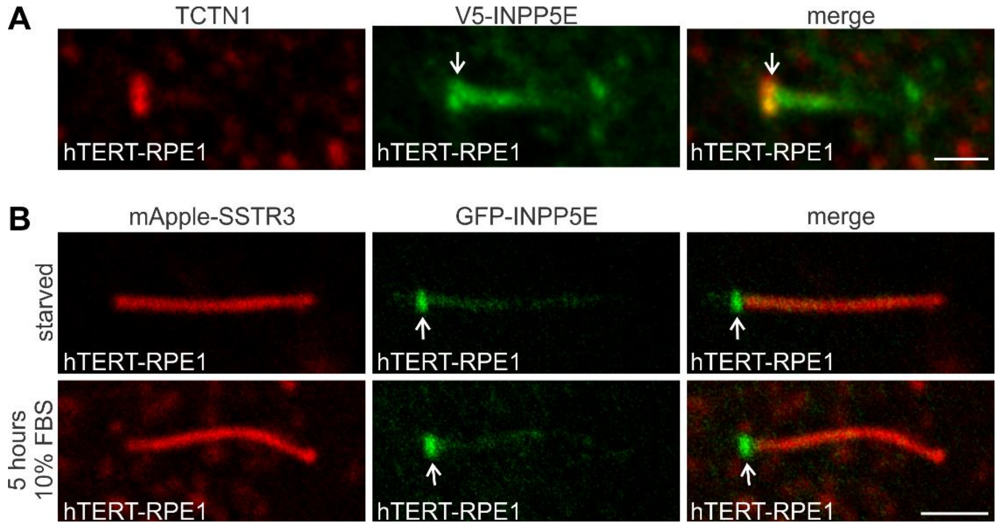


**Figure S3: INPP5E transition zone localization is unaffected by serum stimulation**
**(A)** hTERT-RPE1 cells were transfected with V5-INPP5E, serum starved, fixed with 4% PFA, stained with V5 (green) and TCTN1 (red) antibodies and imaged by confocal microscopy. Arrow indicates transition zone INPP5E localisation, bar indicates 1 µm. Images are representative of n=3 independent experiments.
**(B)** hTERT-RPE1 cells were transfected with mApple-SSTR3 (red) and GFP-INPP5E (green), serum starved, then left untreated or stimulated with 10% FBS for 5 hours and imaged live by confocal microscopy. Arrow indicates transition zone INPP5E localisation, bar indicates 2 µm. Images are representative of n=3 independent experiments.

**References**

Chavez, M., Ena, S., Van Sande, J., De Kerchove D'exaerde, A., Schurmans, S., and Schiffmann, S.N. (2015). Modulation of Ciliary Phosphoinositide Content Regulates Trafficking and Sonic Hedgehog Signaling Output. *Dev Cell* 34**,** 338-350.

Hammond, G.R., Schiavo, G., and Irvine, R.F. (2009). Immunocytochemical techniques reveal multiple, distinct cellular pools of PtdIns4P and PtdIns(4,5)P(2). *Biochem J* 422**,** 23-35.
